# Review of the AlGaN/GaN High-Electron-Mobility Transistor-Based Biosensors: Structure, Mechanisms, and Applications

**DOI:** 10.3390/mi15030330

**Published:** 2024-02-28

**Authors:** Chenbi Li, Xinghuan Chen, Zeheng Wang

**Affiliations:** 1 Department of ICU, 3201 Hospital, Hanzhong 723000, China; 2 The China Electronic Product Reliability and Environmental Testing Research Institute, Guangzhou 510610, China; 3 Manufacturing, CSIRO, Lindfield, NSW 2070, Australia

**Keywords:** biosensor, medical sensor, biomolecule, AlGaN/GaN, HEMT

## Abstract

Due to its excellent material performance, the AlGaN/GaN high-electron-mobility transistor (HEMT) provides a wide platform for biosensing. The high density and mobility of two-dimensional electron gas (2DEG) at the AlGaN/GaN interface induced by the polarization effect and the short distance between the 2DEG channel and the surface can improve the sensitivity of the biosensors. The high thermal and chemical stability can also benefit HEMT-based biosensors’ operation under, for example, high temperatures and chemically harsh environments. This makes creating biosensors with excellent sensitivity, selectivity, reliability, and repeatability achievable using commercialized semiconductor materials. To synthesize the recent developments and advantages in this research field, we review the various AlGaN/GaN HEMT-based biosensors’ structures, operations mechanisms, and applications. This review will help new researchers to learn the basic information about the topic and aid in the development of next-generation of AlGaN/GaN HEMT-based biosensors.

## 1. Introduction

The analysis of biological molecules is a cornerstone in medical diagnostics, a field which is historically reliant on techniques such as enzyme-linked immunosorbent assay (ELISA), immunoturbidimetry, and visual agglutination [1,2,3,4]. These traditional methods, however, are hampered by their need for expensive equipment, specific reagents, and a considerable time investment. In response to these limitations, there has been a rising demand for more efficient and cost-effective biosensors which are capable of detecting, identifying, and quantifying target biomolecules in a range of physiological solutions. Analytes, including enzymes, nucleic acids, and antibodies, induce changes in sensor behaviors such as current, conductance, and temperature. These changes can be precisely measured, offering a window into the complex biochemical interactions occurring within biological samples.

Field-effect transistor (FET)-based biosensors represent a significant advancement in this domain, offering a versatile approach for detecting a broad spectrum of biomolecules, including viruses, nucleic acids, DNA, RNA, and proteins [5,6,7,8]. Renowned for their rapid response times and exceptional sensitivity, FET-based biosensors are a cost-effective solution compared to conventional methods [9]. When operated in the subthreshold regime, where the gate bias is near the threshold voltage, these sensors demonstrate maximum sensitivity due to the drain current’s exponential response to gate voltage fluctuations [10,11,12,13]. The growing popularity of FET-based biosensors in biomolecule detection is a testament to their unique advantages and potential, with ion-sensitive and metal-oxide-semiconductor FETs being the most prominent structures in this category [14].

In recent years, AlGaN/GaN high-electron mobility transistors (HEMTs) have emerged as a focal point in biosensor research, attributed to the exceptional properties of GaN materials and the AlGaN/GaN heterostructure [15,16]. Distinct from silicon-based FETs, AlGaN/GaN HEMTs are characterized by their undoped nature and the formation of a high-density two-dimensional electron gas (2DEG) at the AlGaN/GaN interface, which provide a broad range of devices to be designed [17,18,19]. This formation results from the piezoelectric polarization of the strained AlGaN layer and the spontaneous polarization occurring between the GaN and AlGaN layers and the misfit of the energy bandgap [20,21]. Without the impurity scattering, 2DEG features a high mobility, which can improve the sensitivity of the biosensors. Meanwhile, the proximity of the 2DEG channel to the sensor’s surface significantly enhances its sensitivity to changes in surface conditions [22,23], establishing AlGaN/GaN HEMTs as a superior platform for high-sensitivity biosensing. Furthermore, the robust thermal and chemical stability of GaN materials allows AlGaN/GaN HEMT-based biosensors to function effectively in extreme temperatures and harsh chemical environments, broadening their applicability across a diverse range of molecular detections.

This review is aimed at providing a comprehensive overview of the structures, mechanisms, and diverse applications of AlGaN/GaN HEMT-based biosensors. It delves into the intricate structures and operating mechanisms of HEMT-based biosensors, setting the stage for an exploration of their various applications. These applications span from breast cancer sensors and HER2 detection sensors to the intricate detection of DNA and RNA sequences, and pH sensors, underscoring the versatile and critical role of these advanced biosensors in modern medical diagnostics.

## 2. Structure and Mechanism

AlGaN/GaN HEMT-based biosensors have a similar structure to FET-based biosensors, featuring gate, source, and drain terminals. When biomarkers (e.g., antibodies, viruses, aptamers, enzymes, molecules, or proteins) are introduced into the sensing region (e.g., gate terminal), significant variations in electrical characteristics (e.g., drain current, threshold voltage, drain-to-source voltage drop) of the AlGaN/GaN HEMT can be induced. This occurs because biomarkers impact the 2DEG channel potential, affecting the conductivity of the 2DEG [24]. Consequently, the short distance between the 2DEG channel and the surface enhances the sensitivity of AlGaN/GaN HEMT-based biosensors. Furthermore, due to the polarization effect, AlGaN/GaN HEMTs typically operate in a normally on mode, implying that the gate electrode is not necessary, as the drain and source terminals are electrically connected by the 2DEG. This allows for a wide range of biosensor designs, several of which will be discussed.

**Figure 1:**
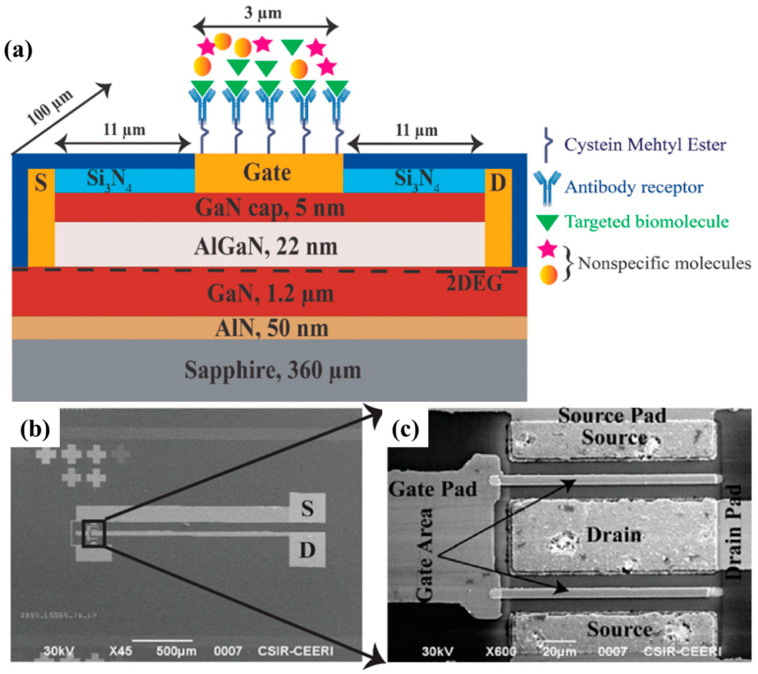
(**a**) Schematic of AlGaN/GaN HEMT-based biosensor with markings of epitaxial structure and device design. (**b**) Scanning electron microscope images of the biosensor. (**c**) Zoomed in area of the active area. Reprinted from reference [25].

Figure 1 shows a biosensor with a common AlGaN/GaN structure [25]. The epitaxial wafer is typically grown by metal oxide chemical vapor deposition (MOCVD), with an Ohmic contact-type source and drain electrodes, and a Schottky contact metal stack at the gate terminal. The gate terminal is divided into two parts: the sensing region (labeled as the gate area) and biasing pads. The 2DEG can be modulated by applying a bias voltage to the gate pad to adjust the sensing sensitivity. Cysteine methyl ester (CME) is formed at the sensing region surface as a bridge between the gate and antibody receptor. The antibody receptor ensures that only targeted biomolecules are detected. When targeted biomolecules are added to the sensing area, the drain current changes due to surface charge alterations, allowing for the detection of targeted biomolecules by monitoring the drain current.

**Figure 2:**
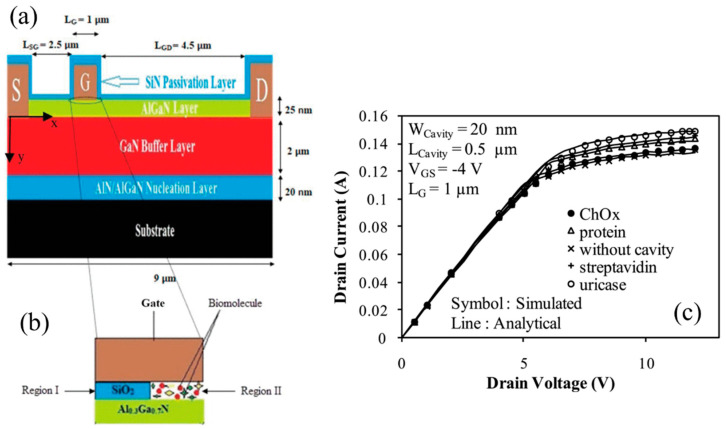
(**a**) Structure of AlGaN/GaN MOSHEMT with nanogap cavity under the gate. (**b**) Expanded view of cavity region introduced below gate region. (**c**) The output curves of simulated and analytical model for C_h_O_x_, protein, streptavidin and uricase biomolecules. Reprinted from reference [26].

Figure 2a shows the structure of an AlGaN/GaN MOSHEMT with a nanogap cavity under the gate, consisting of two regions, as shown in Figure 2b. Part of the gate dielectric is etched laterally, forming a nanogap cavity for biomolecule addition and detection. The channel potential under the nanogap cavity depends on the biomolecules due to their different permittivity values, affecting the drain current. Pal et al. proposed an analytical model for an AlGaN/GaN MOS HEMT-based biosensor using a dielectric modulation approach. When the biomolecule is inserted into the nanogap cavity, the gate capacitance of the GaN HEMT will be changed, which can affect the surface potential. Pal et al. analyzed the relationship between the on-state current and gate capacitance, and a simulation was carried out to confirm the model. Figure 2c exhibits the output curves from the simulated and analytical models for ChOx, protein, streptavidin, and uricase biomolecules [26], showing that the on-state current values differ for each biomolecule. However, this method has limitations, especially in detecting biomolecules with similar permittivity values, such as proteins (permittivity of 2.5) and streptavidin (permittivity of 2.1), where the current curves almost overlap. Moreover, the purity of the detection sample, which can impact permittivity values, is essential in this method.

**Figure 3:**
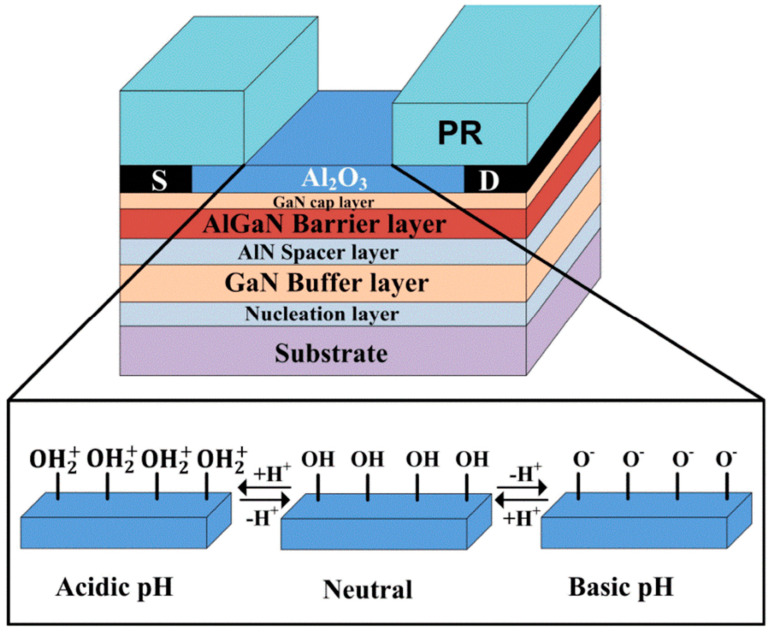
Schematic illustration of AlGaN/GaN HEMT sensing mechanism. Reprinted from reference [27].

It is known that the 2DEG channel naturally exists at the AlGaN/GaN interface due to polarization, allowing AlGaN/GaN HEMT-based biosensors to operate without a gate electrode [27,28]. Figure 3 exhibits a schematic illustration of AlGaN/GaN HEMT pH sensing [27], where Al_2_O_3_ is deposited by the atomic layer deposition technique. This layer serves as both the sensing membrane and the device’s passivation. By doing so, a large binding region and a chemically stable interface are achieved. The surface potential of the sensing region depends on the surface charges, which change by attracting counter-ions when exposed to detection samples. Correspondingly, the density of the 2DEG at the AlGaN/GaN interface increases with positive charge attraction or decreases with negative charge attraction [29]. Therefore, the properties of the sample can be detected by monitoring changes in the output current.

## 3. Applications of AlGaN/GaN HEMT-Based Biosensors

The detection of various biological samples holds paramount importance in the field of medical diagnostics. Identifying interactions between antigens and antibodies, for instance, is crucial in signaling the presence of specific biomarkers associated with viruses, cancers, or other diseases. In this light, the emergence and development of AlGaN/GaN HEMT-based biosensors mark a significant stride in the realm of disease detection, prevention, and health management. These advanced biosensors bring forth a new era in diagnostics, where the early detection and accurate identification of diseases are crucial for effective treatment and management. Their ability to sensitively and specifically detect biomolecules opens up new possibilities in tracking disease progression and evaluating treatment efficacy, potentially transforming the landscape of medical diagnostics.

Moreover, AlGaN/GaN HEMT-based biosensors epitomize the integration of advanced technology into healthcare, offering rapid and reliable detection that is vital for timely intervention. The role these biosensors play extends beyond mere detection; they are instrumental in revolutionizing preventive medicine and patient care. By facilitating an early and precise diagnosis, these biosensors empower healthcare providers with critical information that aids in formulating targeted treatment plans, monitoring patient responses to treatments, and adjusting medical interventions as needed. This not only enhances the quality of patient care but also paves the way for personalized medicine, where treatments are tailored to individual patient profiles based on precise diagnostic data.

### 3.1. Breast Cancer Sensor

Breast cancer remains a major public health issue globally, particularly for women. It is reported that the diagnosis and mortality rates of breast cancer constitute approximately 24.2% and 15.0% of all cancers, respectively. A high mortality rate is often due to late-stage diagnoses [30,31], indicating that early detection can significantly reduce mortality. Traditional diagnostic techniques for breast cancer, such as mammography [32,33], enzyme-linked immunosorbent assay [34], sonography [35], and magnetic resonance imaging [36], are complex and time consuming, hindering high-frequency diagnostics. In contrast, biosensors offer the potential for the rapid and cost-effective diagnosis of breast cancer.

**Figure 4:**
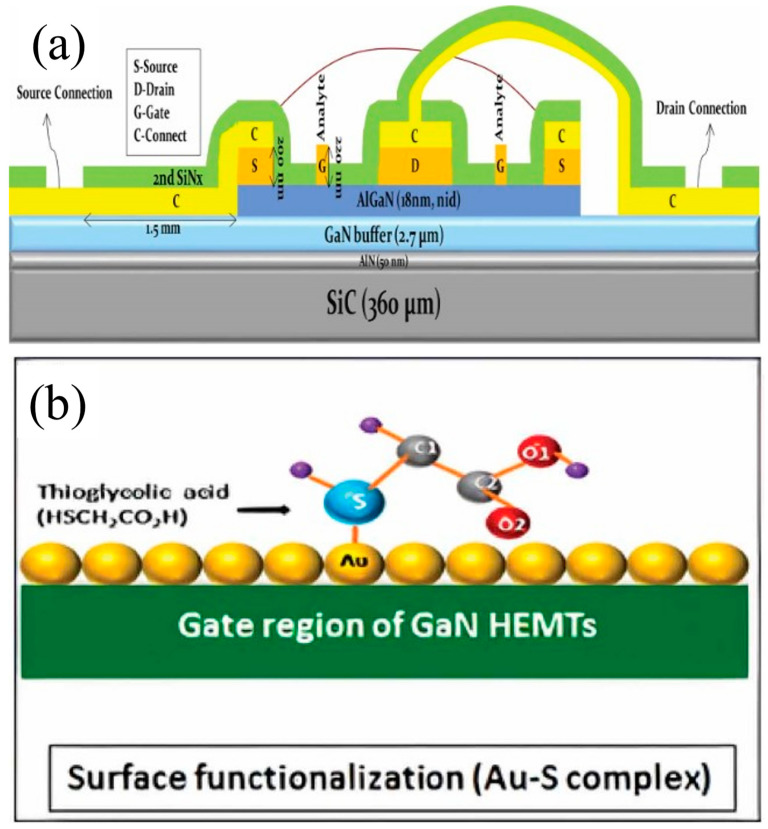
(**a**) Design of GaN HEMT based biosensors. (**b**) Surface functionalization of gold with TGA and antibody. Reprinted from reference [37].

Figure 4a shows a schematic of an AlGaN/GaN HEMT-based biosensor for breast cancer diagnosis [37]. The gate contact, formed by Ni-based Schottky contacts with a top layer of Au, serves as the sensing region. The interaction between antigens and antibodies is the operational principle behind this biosensor. To facilitate this, the sensing region requires a specific design. C-erbB2, a biomarker for breast cancer, non-small cell lung cancer, salivary gland cancer, and others, is selected as the target biomarker for early detection [37,38,39]. The sensing region of the Schottky gate metal is functionalized with thioglycolic acid (TGA). Self-assembled monolayers of TGA and HSCH2COOH, an organic compound with thiol and carboxylic acid functional groups, are anchored on the Au surface, bridging between a specific antibody and the sensing region, as shown in Figure 4b. The interaction between the antigen and antibody alters the potential at the gate area (sensing region), enabling the detection of the breast cancer biomarker C-erbB2 under constant drain bias.

**Figure 5:**
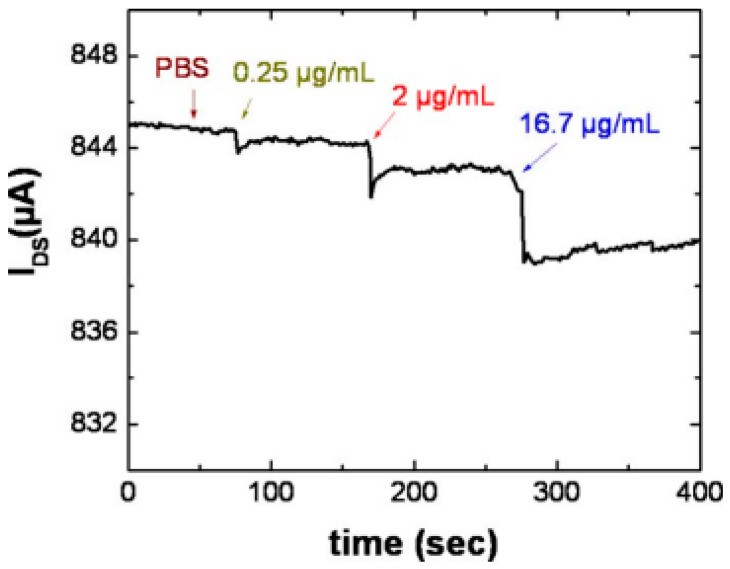
Drain current of an AlGaN/GaN HEMT-based biosensor over time for c-erbB2 antigen from 0.25 to 17 μg/mL. Reprinted from reference [38].

Chen et al. reported on an AlGaN/GaN HEMT-based biosensor using antibody-functionalized Au gates for c-erbB2 antigen detection [38]. Figure 5 displays the time-resolved drain current for c-erbB2 antigen concentrations ranging from 0.25 to 17 μg/mL. This biosensor demonstrates specificity and stability, as evidenced by the absence of current change upon the addition of buffer solution (PBS) at around 50 s. When 0.25 μg/mL c-erbB2 antigen is added to the sensing region, there is a noticeable reduction in drain current. The response time is less than 5 s, indicating the biosensor’s sensitivity and rapid response to the c-erbB2 antigen.

**Figure 6:**
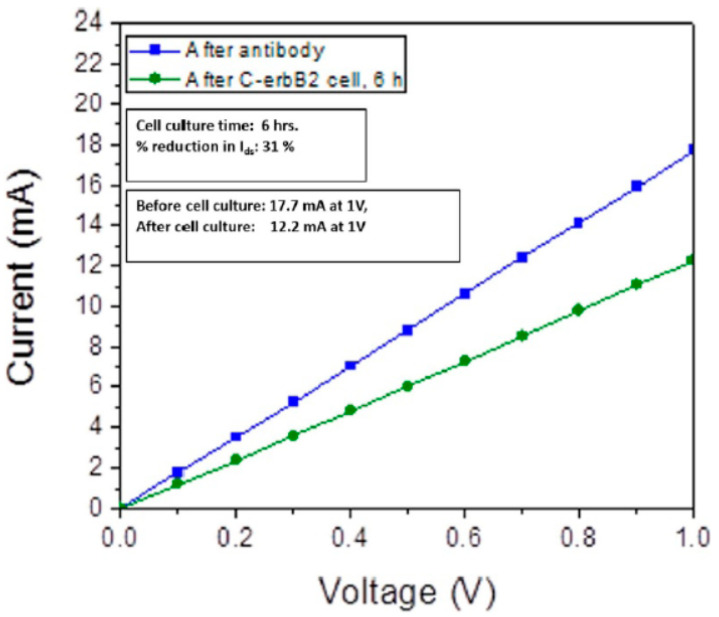
Response of the biosensor after incubation in human cell line positive for breast cancer biomarker C-erbB2. Reprinted from reference [37].

Chaturvedi et al. studied an AlGaN/GaN HEMT-based biosensor for C-erbB2 detection through simulation [37]. The biosensor, designed on a SiC substrate using Silvaco Atlas, shows a significant response to C-erbB2. As depicted in Figure 6, the drain current decreases from 17.7 mA to 12.2 mA after cell culture, with a high sensitivity of 31%, confirming its capability to detect C-erbB2 protein.

### 3.2. HER2 Detection Sensor

The growth of a normal cell is regulated by the human epidermal growth factor receptor-2 (HER2), which is a transmembrane tyrosine kinase receptor. And the cancer can induce the overexpression of HER2 [40,41], which indicates that HER2 can be used as a prognostic, predictive, and therapeutic marker in cancers. Enzyme-linked immunosorbent assay [42,43], immunohistochemistry [25], and fluorescent in situ hybridization [25] are available to detect the overexpression of HER2. However, the above method is time-consuming, requires a strict environment, and is labor intensive. AlGaN/GaN HEMT-based biosensors are suitable for the high-sensitivity and fast-response detection of HER2.

**Figure 7:**
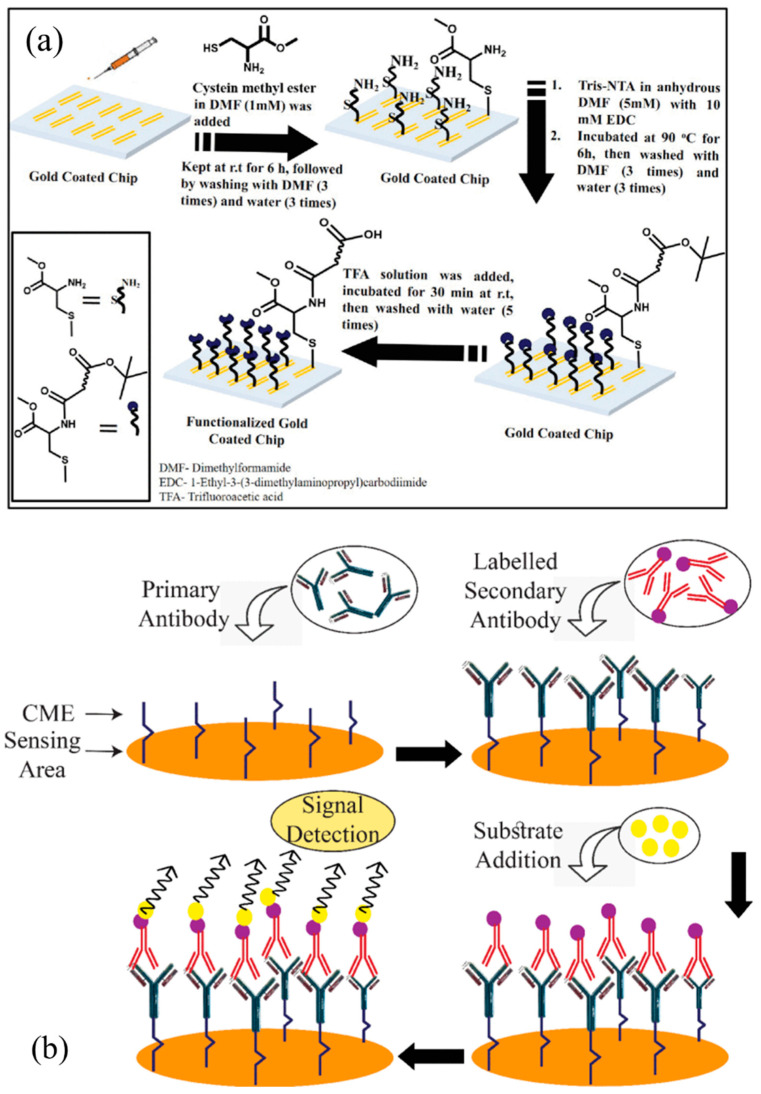
(**a**) Stepwise schematic representation of CME layer formation on the sensing area. (**b**) Stepwise graphical illustration of the detection method. Arrow direction shows the flow of the process. Reprinted from reference [25].

Mishra et al. reported an AlGaN/GaN HEMT-based biosensors to detect HER2 using the enzyme-linked immunosorbent assay (ELIAS) [25]. The structure of the biosensor is shown in Figure 1a, which is discussed in Section 2. Figure 7 exhibits a procedure schematic of the ELIAS method. As shown in Figure 7a, in the first step, the cysteine methyl ester (CME) is formed at the sensing region’s surface as the bridge between gate and antibody receptor, which leaves the open carboxylic group (COOH-) on the top for the anti-HER2 conjugation. Later, the anti-HER2 can be immobilized over the gate region due to the interplay between CME and anti-HER2, which can be confirmed by the ELIAS test, and the bovine serum albumin (BSA) can be used to strengthen the sensor specificity. Confirmation of activation of the sensing region is required to confirm that the anti-HER2 has been successfully immobilized on the surface sensing surface and the anti-HER2 uses the HER-2 antigen which is specific to anti-HER2. Then, the AlGaN/GaN HEMT-based biosensor is ready for the detection of the HER2 antigen. The operation of the HER2 sensor is illustrated in Figure 7b.

**Figure 8:**
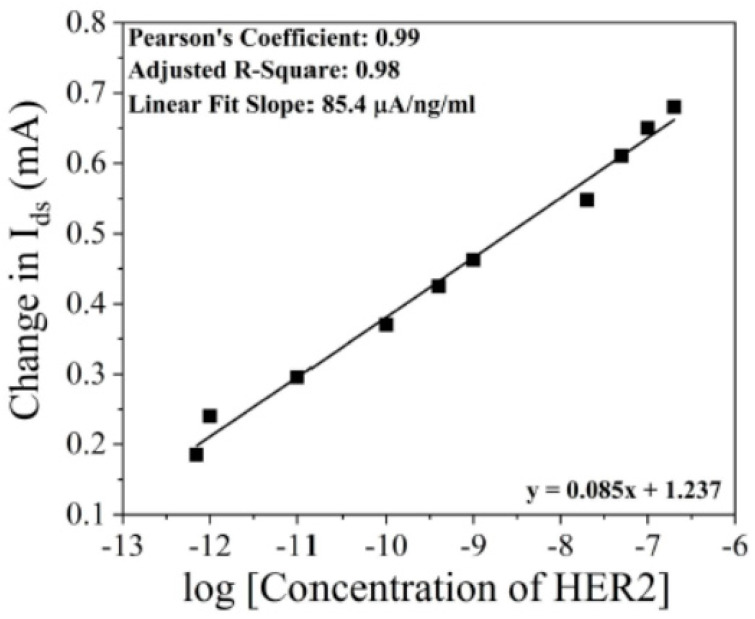
Change in the drain current for concentration of HER2 antigen in the range of 0.7 pg/mL to 200 ng/mL. Reprinted from reference [25].

Figure 8 shows the response of the AlGaN/GaN HEMT-based biosensor to the HER2 by monitoring the drain current, in which the performance of the biosensor is explored over a wide range of HER2 concentrations from 0.7 pg/mL to 200 ng/mL [25]. The drain current increases with the increase in HER2 concentration due to the change in surface potential induced by the HER2 antigen. Furthermore, the relationship between the change in the drain current and HER2 concentrations at the log-scale can be linearly fitted with the standard deviation of 2.05%, which is very useful in the analysis of HER2 detection. And the characteristic sensitivity of the CME-functionalized AlGaN/GaN HEMT for HER2 antigen can be achieved with the curve slope of 85 μA/ng/mL.

**Figure 9:**
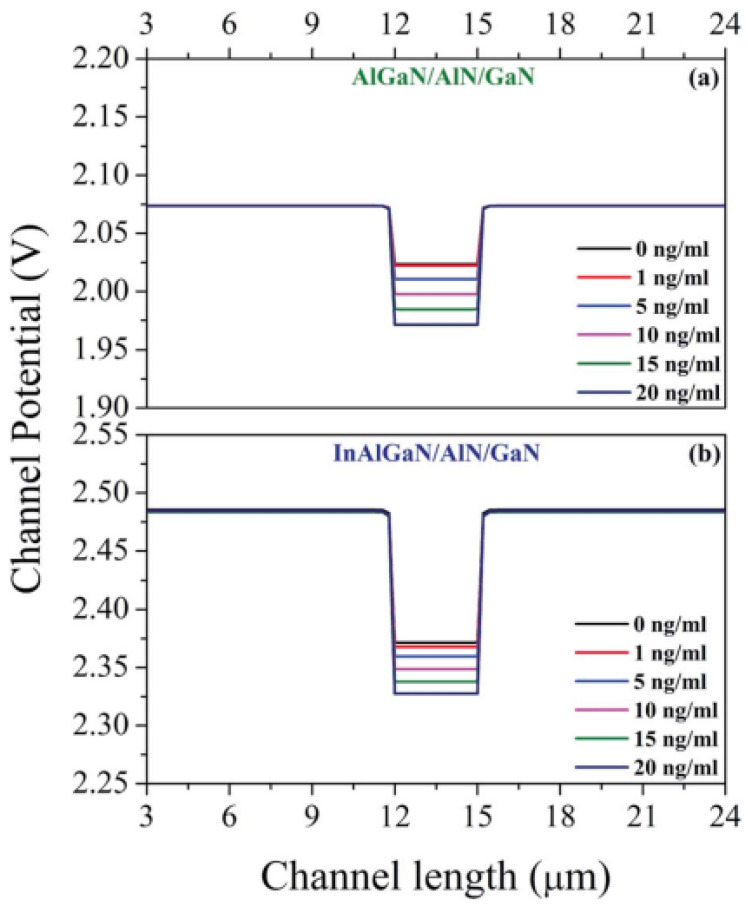
Distribution of the channel’s electrostatic potentials along the channel for the (**a**) AlGaN- and (**b**) InAlGaN-based HEMTs, with different concentrations of HER2 presented. Reprinted from reference [44].

Mishra et al. also studied the performance of the AlGaN/GaN and InAlGaN/GaN HEMTs-based biosensors for HER2 detection using a machine-learning-based model and TCAD simulations [44]. The distributions of channel potential for both types of biosensors with various concentrations of HER2 are shown in Figure 9a and Figure 9b, respectively. The channel barrier increases with the increase in HER2 concentrations, which indicates that negative charges accumulate in the sensing region due to the impact of HER2. As a result, the 2DEG at the heterointerface will reduce with the increase in HER2 concentrations. And the change in channel potential for the InAlGaN-based HEMT is observed to be greater than the AlGaN-based HEMT, which means that the InAlGaN HEMT-based biosensors are more sensitive. However, the InAlGaN HEMTs feature an InN-containing barrier, so the performance of the InAlGaN HEMT-based biosensors will be impacted by the alloy scattering and miscibility [45,46]. The reliability issues are also important due to the interface roughness and acoustic and optical phonons [46].

### 3.3. DNA and RNA Sensor

Nucleic acids, such as deoxyribonucleic acids (DNAs) and ribonucleic acids (RNAs), are critical biomolecules that store, transfer, and express genetic information. Diseases related to viruses and cancers can be diagnosed by detecting specific DNA or RNA sequences associated with these pathogens or malignant cells. The principle of complementary base pairing in nucleic acids provides a theoretical foundation for designing nucleic acid probes.

**Figure 10:**
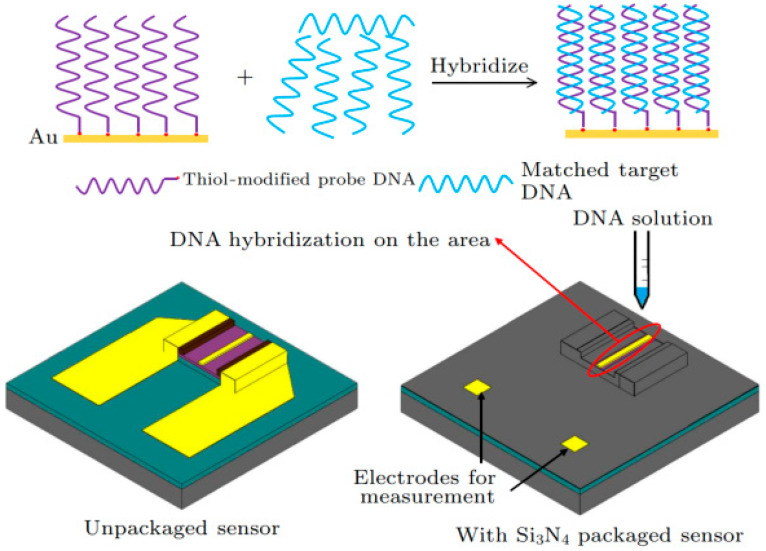
The detection procedure of DNA hybridization on the sensor. Reprinted from reference [47].

Zhang et al. studied Au-gated AlInN/GaN HEMT and AlGaN/GaN HEMT-based biosensors for DNA detection [47]. To detect specific DNA molecules, a thiol-modified probe DNA sequence must be immobilized on the Au surface, as illustrated in Figure 10. In their research, the sequence 5′-HS-(CH2)6-ATACCAGCTTATTCAATT-3′ was synthesized as the thiol-modified probe DNA, enabling the detection of only its complementary target DNA sequence, 5′-AATTGAATAAGCTGGTAT-3′. When the DNA immobilized on the sensing region matches the target DNA, the surface potential changes, allowing for the detection of the target DNA through monitoring variations in drain current.

**Figure 11:**
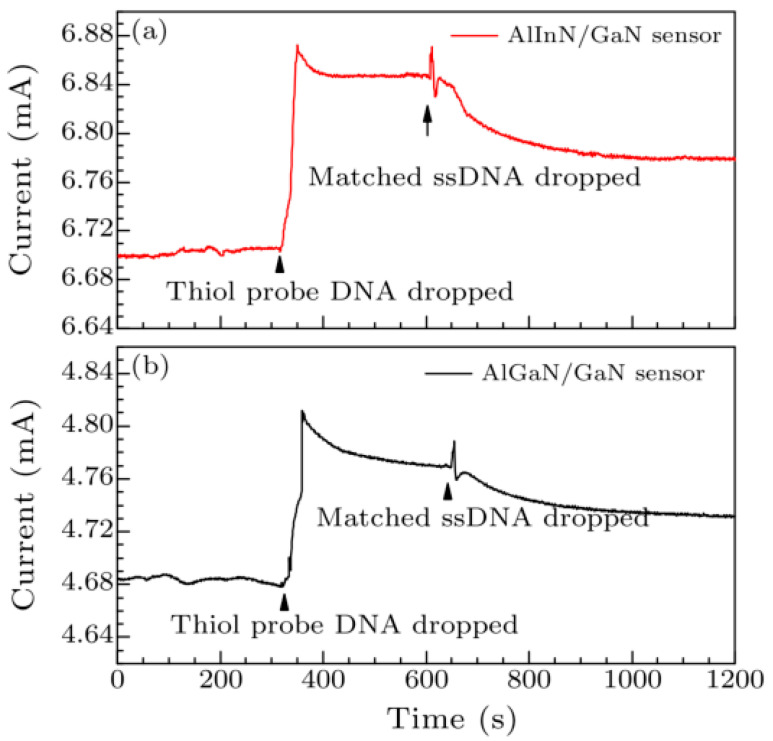
Time dependence of the drain current change in Au-gated (**a**) AlInN/GaN HEMTs-based and (**b**) AlGaN/GaN HEMTs-based biosensors upon exposure to thiol-modified DNA and matched DNA at *V*_DS_ = 0.5 V. Reprinted from reference [47].

Figure 11 presents the time-resolved detection of DNA for both AlInN/GaN HEMT and AlGaN/GaN HEMT-based biosensors using the same concentration and volume of thiol probe DNA solution and target DNA solution. The current change observed in the AlInN/GaN HEMT-based biosensor is greater than that in the AlGaN/GaN HEMT-based biosensor, suggesting that AlInN/GaN may be more suitable for DNA detection sensors. When thiol probe DNA is applied to the Au gate, the drain current rapidly increases due to the formation of Au-S bonds, as Au atoms attract electrons from sulfur atoms [48]. These bonds introduce additional positive charges on the Au gate surface. The positive charges are neutralized by the negatively charged target DNA molecules in the aqueous solution, resulting in a decrease in drain current when the target DNA is added.

It should be noted that the RNA sequence has a similar principle of complementary base pairing in nucleic acids to DNA sequences, so the design and operation of an RNA sensor is same as that for the DNA sensor.

### 3.4. pH Sensor

pH values hold a critical position in the biomedical industry, acting as essential biomarkers for assessing the severity and progression of various diseases. These values, reflective of the acidic or basic nature of a biological environment, can provide key insights into the physiological state of a patient, particularly in conditions where pH imbalance is a known symptom or consequence. The precise measurement and monitoring of pH levels, therefore, are vital in both diagnostics and the effective management of numerous health conditions.

The robust nature of GaN materials comes to the fore in the construction of AlGaN/GaN HEMT-based biosensors, which are particularly suited for pH measurement. GaN’s high thermal and chemical stabilities are crucial properties that enable these biosensors to function reliably under extreme conditions. This resilience is especially important for pH sensors, as they often need to operate in environments with high temperatures or exposure to chemically reactive or corrosive substances. Whether it is within the harsh chemical milieu of industrial processes or the challenging conditions of biological systems, these biosensors maintain their accuracy and sensitivity.

Furthermore, AlGaN/GaN HEMT-based biosensors’ ability to operate in such demanding environments without degradation or loss of functionality ensures consistent and reliable pH measurements. This reliability is essential not only for ongoing patient care but also for experimental and research purposes, where consistent data quality is paramount. Their adaptability and resilience make these biosensors an invaluable tool in a wide array of biomedical applications, ranging from clinical diagnostics to pharmaceutical research, where making precise pH measurements is a critical requirement.

Commonly, an open-gate structure is utilized in the design of pH sensors [27,28], as depicted in Figure 3. The sensing region’s surface can form neutral hydroxyl groups (MOH, where M represents Si or metals). Protonation and deprotonation processes can alter the charges at the sensing surface [49], which are dependent on the pH values, as described in the following reactions [28,50]:

(1) $$MOH↔{MO}^{-}+H^{+}$$

(2) $$MOH+H^{+}↔{MOH}_{2}^{+}$$

**Figure 12:**
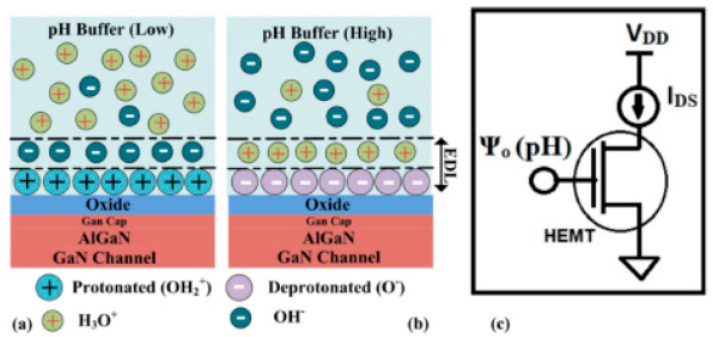
Schematic illustration of charge formation at the oxide/electrolyte interface in (**a**) acidic PBS, (**b**) basic PBS, and (**c**) simplified equivalent model depicting signal transduction for AlGaN/GaN HEMT-based sensor. Reprinted from reference [27].

**Figure 13:**
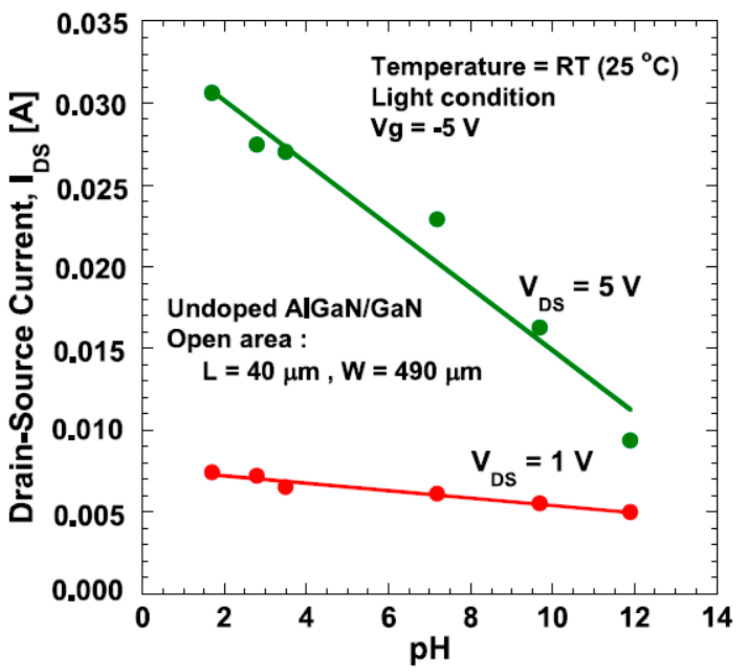
The measured drain current of AlGaN/GaN HEMT-based biosensor under various pH values. Reprinted from reference [28].

In acidic solutions (low pH value), the concentration of H+ is high, leading to the protonation of hydroxyl groups as per Equation (2). This induces the accumulation of positive charges (MOH_2_^+^) at the sensing surface, as shown in Figure 12a. Consequently, the density of the 2DEG increases at low pH values, which is evidenced by the higher drain current in such cases, as illustrated in Figure 13. Conversely, as pH values increase, Equation (1) becomes dominant. The deprotonation of hydroxyl groups induces negative charges (MO^−^), as depicted in Figure 12b, resulting in a linear decrease in the drain current with increasing pH values, as demonstrated in Figure 13.

## 4. Discussion and Overview

AlGaN/GaN HEMT-based biosensors have emerged as a revolutionary technology in the field of biomedical diagnostics, offering unparalleled sensitivity, selectivity, and stability. These biosensors utilize the high electron mobility and direct bandgap of GaN, along with the AlGaN/GaN heterostructure, to provide exceptional performance in detecting various biomolecules, a feature which is critical for early-stage disease diagnosis and management.

The sensitivity of these biosensors is particularly noteworthy. Their ability to detect minute changes in electrical properties in response to biomolecular interactions at the sensing surface is vital for the early detection of diseases. This sensitivity is further enhanced by the proximity of the two-dimensional electron gas (2DEG) to the sensor surface, enabling the detection of low biomolecule concentrations, which is crucial for early disease diagnosis. The selectivity of these biosensors is achieved through careful surface functionalization, allowing for specific binding to target biomolecules. This specificity is essential for accurate disease identification, as demonstrated in the detection of specific biomarkers like C-erbB2 in breast cancer sensors, where thioglycolic acid (TGA) and antibody receptors are utilized.

In terms of stability and reliability, AlGaN/GaN HEMT-based biosensors stand out. Their robustness against high temperatures and harsh chemical environments makes them suitable for various applications, including continuous monitoring and long-term diagnostic use. This stability, combined with their repeatability, renders these biosensors highly desirable in medical and industrial settings. However, the surface and buffer traps can induce the dynamic threshold voltage and on-state current of AlGaN/GaN HEMTs, which will impact the accuracy of the biosensors [51,52,53]. And models of the dynamic threshold voltage and on-state current are required to calibrated detection results of AlGaN/GaN HEMTs-based biosensors [54].

These biosensors have shown significant promise in disease detection, particularly in the early detection of breast cancer, HER2, and various DNA and RNA sequences related to cancers and viruses. Their sensitivity in detecting HER2, a key biomarker in cancer, is a testament to their potential in guiding treatment decisions and monitoring therapy effectiveness. Advancements in DNA and RNA sensing using these biosensors mark a substantial step in genetic diagnostics. Their ability to detect complementary DNA and RNA sequences opens new possibilities in understanding genetic diseases and developing personalized medicine. The specificity and rapid response of these biosensors make them invaluable in genetic research and diagnostics.

The application of these biosensors in pH sensing illustrates their versatility. Accurate and reliable pH sensing is crucial in numerous biomedical and environmental monitoring applications, with direct implications for understanding disease severity and progression.

Despite the advantages, challenges remain, including the need for the miniaturization and integration of these biosensors into portable devices for point-of-care testing. This would significantly broaden their application, especially in remote or resource-limited settings. Further enhancing selectivity and reducing interference from non-target substances are other areas for development. Advanced surface functionalization techniques and the integration of machine learning for data analysis could overcome these challenges, leading to more precise and efficient biosensors.

In short, AlGaN/GaN HEMT-based biosensors represent a significant stride in biosensing technology. Their broad range of applications in medical diagnostics and environmental monitoring, coupled with their high performance, positions them as pivotal tools in advancing personalized medicine and public health. As research continues to evolve in this domain, these biosensors are poised to revolutionize disease detection and management, contributing significantly to healthcare and environmental monitoring.

## 5. Conclusions

This review not only confirms the efficacy of AlGaN/GaN HEMT-based biosensors but also underscores their transformative role in medical diagnostics. Exhibiting exceptional sensitivity, selectivity, reliability, and repeatability, these biosensors stand out for their ability to operate under extreme thermal and chemical conditions. Their robustness makes them indispensable tools in the detection of a wide array of biomolecules, such as c-erbB2, HER2, DNA, and RNA, which are crucial in the early diagnosis of virus-related diseases and cancers. The comprehensive analysis of their applications in detecting breast cancer, HER2, and various nucleic acids, along with pH sensing, showcases their rapid response and superior sensitivity.

The potential of AlGaN/GaN HEMT-based biosensors goes beyond conventional diagnostics. They represent a significant leap forward in enabling the early and accurate detection of diseases, a key factor in improving patient outcomes. The integration of these biosensors into healthcare systems is poised to revolutionize not only how diseases are detected but also how they are managed and prevented. With their advancement, there is a promising horizon for personalized medicine, where diagnostic processes are not just reactive but also proactive and tailored to individual patient needs.

In conclusion, the development and refinement of AlGaN/GaN HEMT-based biosensors mark pivotal advancement in the field of medical diagnostics. Their contribution extends from enhancing the accuracy of disease detection to reshaping preventive healthcare strategies. As research and technology continue to evolve, these biosensors are expected to play an increasingly critical role in disease management, heralding a new era of healthcare in which precision and early intervention are paramount.

## Data Availability

Data are contained within the article.
